# Anticipatory Grief Among Family Caregivers of Cancer Patients in Home Palliative Care: A Constructivist Grounded Theory

**DOI:** 10.3390/healthcare14152312

**Published:** 2026-07-31

**Authors:** Camila Carreira, Ana Ramos, Maria João Mateus, Paula Pinto, Amira Mohammed Ali, Feten Fekih-Romdhane, Selma Yıldırım, Murat Yıldırım, Carlos Laranjeira

**Affiliations:** 1 School of Health Sciences, Polytechnic University of Leiria, Campus 2, Morro do Lena, Alto do Vieiro, Apartado 4137, 2411-901 Leiria, Portugal; 5240238@my.ipleiria.pt (C.C.); ana.claudiaeramoss@gmail.com (A.R.); 2 Baixo Vouga Community Palliative Care Team, Local Health Unit of the Region of Aveiro, 3750-154 Águeda, Portugal; 12817@ulsra.min-saude.pt (M.J.M.); 74258@ulsra.min-saude.pt (P.P.); 3 Department of Psychiatric Nursing and Mental Health, Faculty of Nursing, Alexandria University, Smouha, Alexandria 21527, Egypt; amiramali@alexu.edu.eg; 4 Faculty of Medicine of Tunis, Tunis El Manar University, Tunis 1068, Tunisia; feten.fekih@gmail.com; 5 The Tunisian Center of Early Intervention in Psychosis, Department of Psychiatry, Ibn Omrane, Razi Hospital, Tunis 2010, Tunisia; 6 Graduate School of Natural and Applied Sciences, Kafkas University, Kars 36100, Türkiye; 245266003@ogr.kafkas.edu.tr; 7 Department of Psychology, Faculty of Science and Letters, Ağrı İbrahim Çeçen University, Ağrı 04100, Türkiye; muratyildirim@agri.edu.tr; 8 Psychology Research Center, Khazar University, Baku 1009, Azerbaijan; 9 Centre for Innovative Care and Health Technology (ciTechCare), Polytechnic University of Leiria, Campus 5, Rua das Olhalvas, 2414-016 Leiria, Portugal; 10 Comprehensive Health Research Centre (CHRC), University of Évora, 7000-801 Évora, Portugal

**Keywords:** anticipatory grief, family, caregivers, palliative care, home care, loss, qualitative study

## Abstract

**Background/Objectives**: Anticipatory grief is a significant phenomenon in the experience of family caregivers of individuals receiving palliative care (PC) at home, representing a complex emotional response to impending loss. Nevertheless, there is scarce research supporting an in-depth, context-sensitive understanding of caregivers’ lived experiences in home settings. This study aims to develop a grounded theory that explains how family caregivers of individuals receiving PC at home experience anticipatory grief. **Methods**: This qualitative study used a constructivist grounded theory approach. Fifteen family caregivers of cancer patients receiving PC were recruited from two Portuguese-specialized palliative care teams. Data collection took place from January to April 2026 through semi-structured, in-depth interviews. Participants were purposively sampled to ensure variation in experiences. All interviews were audio-recorded and transcribed verbatim. Data analysis was conducted in line with grounded theory, using the constant comparative method. Theoretical saturation was achieved after 15 interviews. The COREQ checklist was followed. **Results**: A theory was built around the core category “(Re)constructing meanings in the face of imminent loss in the context of palliative caregiving”, from which three categories emerged: (1) between presence and loss: life on hold; (2) challenges in the caregiving relationship; and (3) assigning meaning and purpose to the anticipation of death. **Conclusions**: Overall, the findings conceptualize anticipatory grief as a dynamic and evolving process of meaning reconstruction, offering a theoretical framework to inform more nuanced and context-sensitive support for family caregivers.

## 1. Introduction

At the global level, an estimated 73.5 million people require palliative care (PC) each year, although only about 14% have effective access to these services [1]. This gap is largely due to the scarcity of specialized PC services, particularly in low- and middle-income countries [2]. In Portugal, between 60% and 75% of people near the end-of-life (EoL) could benefit from PC (approximately 63,000 to 80,000 individuals per year); however, only 12% to 15% actually receive such care, highlighting a significant deficit in the healthcare system’s response [3].

PC constitutes an active, holistic, and person-centered approach aimed at individuals of all ages experiencing significant suffering related to serious illness. Its primary goal is to improve the quality of life of patients, as well as their families and caregivers [4], by not only alleviating pain and other physical symptoms, but also addressing the psychosocial and spiritual needs associated with illness. According to Nyatanga [5], PC aims to ensure a life with quality until the end, while also promoting a dignified and individualized death. Care is delivered by multidisciplinary teams involving professionals from different fields, working in an integrated manner to: (1) maximize quality of life; (2) relieve pain and other physical symptoms; (3) provide psychosocial and spiritual support; and (4) support the family throughout the course of the illness and during the grieving process [4].

Receiving a diagnosis of a life-threatening illness, as well as becoming aware of its progression and the potential proximity of death, constitutes a profoundly impactful experience for family members, significantly affecting their psychological balance. This impact is particularly relevant in the context of PC and is considered one of the main aspects to be addressed in family-focused interventions [6]. In this regard, community PC services play a crucial role by offering continuous and holistic support within the home environment, where most caregiving takes place [7]. Through regular assessment, psychosocial support, and timely interventions, these services can help family caregivers manage emotional distress, develop coping strategies, and adapt to the challenges associated with disease progression and impending loss [7,8].

Among life-threatening illnesses, cancer stands out as one of the leading causes of suffering and mortality, with a growing incidence over the years [9,10]. However, beyond oncological diseases, other chronic and advanced conditions—such as cardiovascular, respiratory, progressive neurological diseases, and dementia—also contribute significantly to suffering, functional decline, and mortality, reinforcing the need for integrated PC [11]. Faced with a terminal diagnosis and the need for EoL care, patients commonly rely on the support of a family member or close friend, who gradually assumes the role of caregiver [12]. Informal caregivers are thus individuals who provide care, as well as emotional and social support, without any formal remuneration. However, assuming this role may have significant consequences for the caregiver, affecting not only their well-being but also the quality of the relationship with the care recipient [13]. In this context, family members are often confronted with the progressive deterioration of the patient’s condition, the loss of shared plans and future expectations, and uncertainty regarding the course of the illness. The multiple changes throughout the course of a disease are often associated with high levels of emotional distress, contributing to the development of psychological and mental health problems during the illness and after death [14].

Grief is a universal experience characterized by a set of psychological processes that enable the gradual acceptance of the loss of a loved one and the alleviation of pain through the reorganization of emotional investment across different areas of life [6]. It can be understood as an emotional response to the loss of a person or something of significant emotional value to the individual [15]. The experience of grief is, however, influenced by multiple factors, particularly the quality of family relationships. According to Delalibera et al. [16], openness in communication and cohesion among family members plays a decisive role in facilitating the adaptation process [16]. Thus, healthy family functioning during the caregiving phase—and especially during bereavement—is essential for promoting psychological well-being. Notably, grief is neither linear nor uniform but marked by diverse emotions and individual trajectories that vary depending on personal circumstances and life history. Consequently, the need for support from family members tends to be equally unique, requiring individualized assessment and context-sensitive responses [17].

Anticipating loss may function as a form of emotional preparation that helps reduce the suffering associated with the death of a loved one [18,19]. In PC, anticipatory grief is widely recognized as part of the ongoing grieving process [20]. It refers to the emotional responses that emerge in anticipation of an expected loss and involve grief reactions, psychological adaptation, future planning, and the reorganization of personal life in response to the impending loss [21,22]. Consequently, anticipatory grief may have significant effects on a caregiver’s well-being, influencing their physical, psychological, cognitive, and emotional health. Common manifestations include insomnia, headaches, anxiety, irritability, difficulty concentrating, depression, and, in severe cases, suicidal ideation [23]. Despite these challenges, anticipatory grief may also have adaptive value by fostering emotional preparedness and facilitating the development of coping strategies to manage the changes associated with an inevitable loss [24]. These findings underscore the need for PC services to assess and address anticipatory grief among family caregivers throughout the illness trajectory.

According to clinical practice guidelines in PC, support for family caregivers should be provided continuously throughout the course of a life-threatening illness and the grieving process [25,26]. Identifying those caregivers who are most in need and most likely to benefit from support is an essential component of preventive intervention [27]. Family members experiencing anticipatory grief may struggle to balance the increasing demands of caregiving with attention to their own needs [28]. This growing caregiving burden tends to generate feelings of loneliness and isolation, as family members often withdraw from social contexts and neglect their relational needs [29]. Family caregivers frequently exhibit symptoms of anticipatory grief and psychological distress, often associated with inadequate preparation for death. They tend to present higher levels of anxiety and depression, a greater prevalence of health problems, and poorer bereavement outcomes, negatively impacting their quality of life [30]. Anticipatory grief may also contribute to the reconfiguration of family dynamics, as caregivers assume new roles and responsibilities within the caregiving context [31]. This process entails a progressive transformation of the relationship with the care recipient, marked by the loss of a previously shared identity and the perception of a gradually fading shared future.

Despite the recognition of anticipatory grief as a relevant phenomenon in PC, the available scientific evidence remains limited, particularly regarding qualitative studies focusing on the experiences of family caregivers in home settings [32,33,34,35]. This gap hinders a deeper understanding of the emotional impact of anticipatory grief during the caregiving period and, consequently, limits the development of well-grounded preventive interventions. The present study aims to develop a grounded theory that explains how family caregivers of individuals receiving PC at home experience anticipatory grief. It is expected that this study will contribute to the early identification of the effects of anticipatory grief, as well as to the development of strategies to prevent its progression into more complex and prolonged forms.

## 2. Materials and Methods

### 2.1. Study Design

This is a qualitative study grounded in constructivist grounded theory (CGT), as proposed by Kathy Charmaz [36]. This approach differs from classic versions of grounded theory by moving away from a positivist framework. It assumes that both reality and the research process itself are social constructions shaped by context and by the interactions established between the researcher and participants [37]. This methodological choice is aligned with an ontological perspective that views the individual as a subjective, unfinished, and unique being, whose experience must be understood in its complexity and within its context.

To ensure transparency and methodological rigor in this qualitative study, the COREQ (Consolidated Criteria for Reporting Qualitative Research) checklist guidelines [38] were followed (Supplementary Table S1).

### 2.2. Setting and Participants

Participants were recruited through contacts from two specialized PC teams in the central region of Portugal. The sample consisted of family caregivers of cancer individuals receiving PC in a home setting. The following inclusion criteria were defined: (1) being 18 years of age or older; (2) having served as the primary family caregiver for at least three months, providing care at home; and (3) having the ability to understand and communicate in Portuguese. Caregivers presenting moderate to severe cognitive impairment, as assessed by the Short Portable Mental Status Questionnaire (SPMSQ; score of ≥5 or a score of ≥6 or higher for those with only a grade school education) [39], were excluded.

In line with the principles of Charmaz’s CGT, a progressive sampling strategy was adopted [34]. In the initial phase, the first 10 participants were selected through purposive sampling, aiming to ensure the inclusion of caregivers with relevant experience of the phenomenon under study. Subsequently, theoretical sampling guided the selection of the remaining five participants, enabling the further development, refinement, and saturation of the emerging categories as analysis progressed. This means that participant recruitment continued until theoretical saturation was reached—that is, the point at which the collection of new data no longer contributed meaningful information to the development of analytical categories. The final sample comprised 15 family caregivers.

### 2.3. Data Collection

Data collection was conducted between January and April 2026. Recruitment was carried out in collaboration with healthcare professionals from specialized PC teams, who acted as facilitators in identifying potential participants. Following this initial identification, caregivers were approached either by these professionals or by the research team and were provided with detailed information about the study’s objectives and procedures. Those interested in participating were subsequently contacted to clarify any questions and to schedule the interview.

Data were collected through individual semi-structured interviews, lasting between 30 and 70 min (average of 45 min). All interviews were conducted in person by the principal investigator, a cisgender woman with two years of professional experience as an occupational therapist. All interviews were audio-recorded and later fully transcribed verbatim. Field notes were maintained alongside interviews to document contextual observations, interactions, emotional nuances, and reflexive insights, contributing to the constant comparative analysis and the conceptualization of phenomenon.

Interviews were conducted in participants’ homes or in another location of their choosing, ensuring appropriate conditions of privacy, comfort, and tranquility. During the interviews, various rapport-building strategies were employed, including active listening, the use of open-ended questions, summarizing, paraphrasing, and the management of silence, fostering an ethical, empathetic, and respectful interaction. The principal investigator received specific training in communication techniques for conducting qualitative interviews, ensuring a sensitive approach tailored to the nature of the phenomenon under study.

Prior to the interview, a brief sociodemographic questionnaire was administered to characterize participants (age, gender, marital status, education level, employment status, relationship to the care recipient, living with care recipient, duration of care, family and social support). This was followed by the use of an interview guide developed based on scientific evidence [27,34], which covered five topics: (1) caregiving experience; (2) relationship with the person receiving PC; (3) anticipation of loss; (4) needs and support; and (5) personal changes (Appendix A). The interview began with the following open-ended question: “I would like you to tell me about how you have been experiencing caring for your family member at this stage of life,” followed by additional questions such as: “How has your relationship with your family member been during this time?”; “Have you thought about the possibility of losing your family member?”; and “How have you been experiencing that?”. Moreover, probing questions (e.g., “Can you tell me more?”, “How did you feel?”, “What happened next?”) were used to elicit deeper insights and clarify participants’ experiences.

The interview guide was pre-tested by two experts with advanced training in PC and experience in qualitative research, with the aim of validating the clarity, structure, and sequencing of the questions, allowing for the identification and correction of any potential ambiguities. Each participant was interviewed only once, and transcripts were not returned to participants for validation. This decision was made to minimize participant burden during a period characterized by substantial caregiving responsibilities and emotional distress associated with the progression of the patient’s illness.

### 2.4. Data Analysis

CGT was developed by Kathy Charmaz and involves a set of progressive and interconnected coding stages [36]. The process begins with initial coding, characterized by a line-by-line analysis of participants’ accounts and comparisons to identify similarities and differences between codes (both within individual interviews and across different interviews addressing similar phenomena). This stage allows for the identification of meanings, emotions, actions, and consequences, with particular emphasis on underlying processes [36,40]. This is followed by focused coding, which is more selective and analytically oriented, aiming to synthesize and interpret larger segments of data through the use of the most significant or frequently occurring codes. At this stage, the researcher evaluates the consistency and analytical relevance of the codes in a non-linear process that may involve returning to initial coding whenever new concepts or perspectives emerge [34]. Axial coding deepens the analysis by exploring relationships between categories and subcategories, allowing the identification of connections among the reported experiences and an understanding of reality as socially constructed by participants. This process involves considering context, social conditions, and the meanings attributed to experiences, contributing to a more dense and integrated analysis [36,40]. Finally, theoretical coding consists of integrating the codes and categories into a coherent conceptual framework, resulting in a more comprehensive analytical narrative. This stage should be conducted with caution, avoiding overly objectivist interpretations, and is primarily used as a tool to clarify, deepen, and articulate meanings emerging from the analysis [36].

All interviews were coded by the first and last authors, and agreement was achieved through team discussions. Data storage and management were carried out using the qualitative data analysis software WebQDA (Version 3.0, University of Aveiro, Aveiro, Portugal).

### 2.5. Rigor

Qualitative rigor was anchored in four key dimensions: (1) credibility, (2) originality, (3) resonance, and (4) usefulness. Each dimension was supported by specific practices that strengthened the methodological and interpretive quality of the study [41]. First, credibility refers to the analytical rigor of the study and the extent to which the developed theory is firmly grounded in sufficient and relevant empirical data. This was ensured through constant comparison, theoretical sampling, and systematic coding (initial and focused), as well as the use of analytical memos and the ongoing researcher reflexivity. In addition, credibility and analytical depth were enhanced through member checking and peer debriefing, supporting a more robust, transparent, and critically examined interpretive process.

Consistent with qualitative research principles, reflexivity was actively maintained by the research team as a means of enhancing methodological rigor and clearly identifying the interpretive lenses informing the analysis [42]. The first author (C.C.), a female occupational therapist with two years of experience in gerontology, led the study. The second and third authors (A.R. and M.J.M.)—a gerontologist and a nurse, respectively—possess advanced training in palliative care. The fourth author (P.S.), a clinical psychologist, contributed expertise grounded in palliative care practice. The final author (C.L.) served as the scientific supervisor of the project, bringing extensive clinical and pedagogical experience in palliative care, as well as recognized expertise in qualitative research. Additionally, the other authors (A.M.A., S.Y., F.F.-R. and M.Y.) contributed as scholars in mental health and psychology, supporting the critical and interpretive dimensions of the analysis.

Next, originality refers to the study’s capacity to generate innovative theoretical contributions [41]. It was fostered through progressive conceptual abstraction, the exploration of variations in the data, and the integration of emerging categories, enabling the development of new concepts and the reconceptualization of the phenomenon of anticipatory grief.

Resonance refers to the theory’s ability to meaningfully represent participants’ experiences [41]. It was strengthened through the use of rich illustrative quotations, the preservation of participants’ narrative meanings, and careful attention to emotional expressions and contextual nuances, ensuring that participants can recognize themselves in the interpretive findings.

Finally, usefulness concerns the analytical and practical value of the theory [41]. This was enhanced by clarifying social processes, linking findings to clinical practice in palliative care, and identifying implications for intervention and future research, thereby extending the study’s relevance.

### 2.6. Ethics

The study adhered to the principles of the Declaration of Helsinki and received approval from the Ethics Committee of the Local Health Unit of the Leiria region (Reference No. 120/2025, 12 December 2025). Written informed consent was obtained from all participants before data collection, and they were informed that they could withdraw from the study at any time without consequence. Participation was voluntary and did not involve any form of financial compensation or other benefits. Participants’ identities were protected through the use of alphanumeric codes (P1, P2, …), ensuring anonymity and confidentiality. In cases where participants exhibited signs of emotional distress during the interview, referral for appropriate psychological support was ensured.

## 3. Results

### 3.1. Sample Description

Fifteen family caregivers participated, aged between 63 and 79 years (M = 70.53; SD = 4.47), with a predominance of female caregivers (n = 12) (Table 1). Regarding marital status, most participants were married (n = 13). In terms of educational level, there was a greater representation of individuals with primary education (n = 5) and higher education (n = 5). Most participants were retired (n = 12). Concerning their relationship with the care recipient, spouses were the most common (n = 10), and most participants shared a household with the care recipient (n = 14).

Most participants had been providing care for more than 12 months (n = 13), and reported having both family support (n = 10) and social support (n = 10). It is noteworthy that all care recipients had been diagnosed with advanced-stage cancer at the time of data collection.

### 3.2. Qualitative Findings

The data obtained from the interviews were organized around a core category—(Re)constructing meanings in the face of imminent loss in the context of palliative caregiving—from which three categories emerged: (1) between presence and loss: life on hold; (2) challenges in the caregiving relationship; and (3) assigning meaning and purpose to the anticipation of death.

The core category refers to the dynamic and ongoing process through which family members seek to understand, reinterpret, and make sense of the experience of accompanying a loved one at the end of life while confronting the anticipation of their loss. In this context, anticipatory grief is not viewed as a static state but as an active process of meaning-making, in which family members oscillate between the presence of the care recipient and the growing awareness of their finitude. This reconstruction of meaning is reflected in how they reinterpret time (often experienced as suspended or intensified), redefine the caregiving role, reorganize emotional bonds, and attempt to find purpose or value in the lived experience.

**Figure 1:**
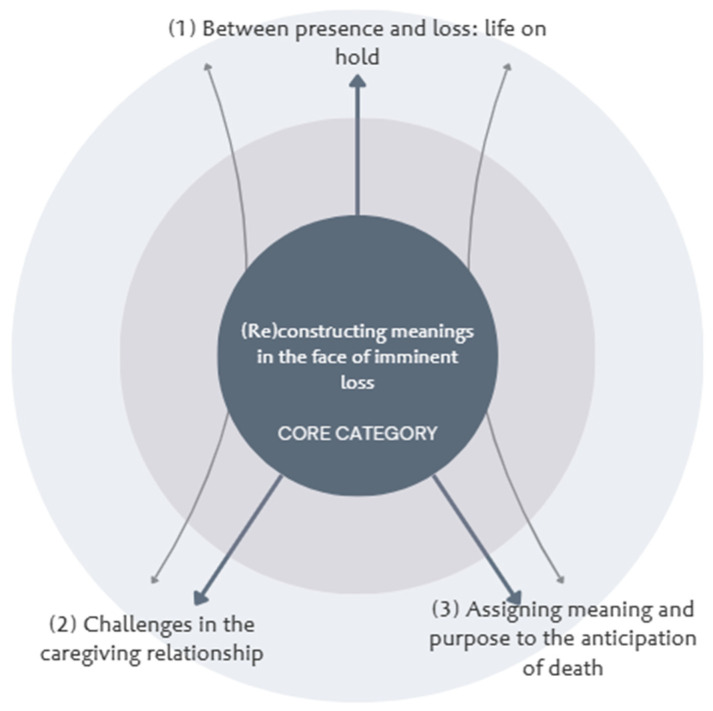
Representations of phenomenon “(Re)constructing meanings in the face of imminent loss in the context of palliative caregiving”.

The different categories and subcategories are presented in Figure 1. Their description will be illustrated with representative excerpts from participants’ accounts, highlighting their empirical grounding and the richness of the reported experiences.

#### 3.2.1. Between Presence and Loss: Life on Hold

Regarding the first category, four subcategories were identified: (1) changes in daily life; (2) ambivalence between lack of time and “small moments” of self-care; (3) coping with caregiving burden; and (4) emotional ambivalence (sadness related to loss vs. relief related to the end of suffering).

(1) Changes in daily life

Changes in daily life constitute a relevant dimension of caregivers’ experiences. Participants report significant alterations in their routines, particularly a loss of autonomy and limitations to previously performed activities. In addition, there is an increased need to remain at home to provide continuous care, contributing to restricted personal freedom and the reorganization of everyday life.

*(P14): “I no longer have the freedom to do the things I used to. I used to go out, take walks; now I can’t, now I can’t do any of that… So I’ve lost the freedom I once had.”*

*(P7): “It’s a kind of dependency because I can’t go out or take care of the things I used to enjoy. Now I only get a few moments here and there. It’s very limiting.”*

*(P4): “I can’t leave the house. If I do, I have to ask someone—my sisters—to stay with her (my mother) while I’m gone. Sometimes I go out briefly to get bread or do some shopping, but always in a rush, always worried about leaving her alone.”*

(2) Ambivalence between lack of time and “small moments” of self-care

This subcategory refers to the ambivalence experienced by caregivers between having little to no time for themselves and valuing small moments of self-care. Participants highlight the difficulty of balancing caregiving demands with attending to their own needs, revealing an ongoing tension between duty and personal well-being. Nevertheless, they recognize the importance of occasional moments of rest and self-care, which play a meaningful role in maintaining emotional balance.

*(P14): “I only leave the house to deal with urgent matters; otherwise, I don’t go out at all. I don’t know what it’s like to go out with a friend, to have a bit of time to talk or chat. Since I became a caregiver, that’s no longer part of my life.”*

*(P6): “Everything has changed—I’ve lost my free time… Since he got sick, I’ve lost my moments of self-care, like reading or going to the movies.”*

*(P9): “I’ve stopped going out. Being at home all the time, I don’t feel like getting ready anymore. It’s been a long time—years, maybe—since I bought any clothing. I used to enjoy dressing well; it made me feel good. I try to take care of myself, but it’s only the basics now…”*

(3) Coping with caregiving burden

Caregiving burden emerges as a significant dimension of the caregivers’ experience, marked by physical and emotional strain resulting from the continuous demands of caregiving. Participants’ accounts reflect fluctuations in mood associated with the intensity and persistence of caregiving responsibilities, as well as a perceived loss of autonomy, expressed as difficulty attending to their own needs due to the centrality of the care recipient.

*(P12): “The responsibility is constant—there are no breaks—and that really affects my state of mind. I feel like I’ve lost control over my own life—everything revolves around him. Many times I know I need to rest or do something for myself, but I simply can’t, because there’s always something more urgent. It’s as if I’ve been pushed into the background.”*

*(P2): “(…) there’s greater wear and tear, both physical and emotional, because things become more complicated as the illness progresses.”*

Additionally, the concentration of responsibilities on a single caregiver intensifies feelings of pressure and ongoing concern, reinforcing the experience of burden. In this regard, P13 noted: *“(…) I’m always worried. I do have another sister, but she’s not here, and if it were shared between us, it would be easier. All the responsibility falls on me…”*

(4) Emotional ambivalence (sadness related to loss vs. relief related to the end of suffering)

Emotional ambivalence is a salient dimension in caregivers’ experiences. Participants express the coexistence of contradictory emotions, revealing sadness associated with the anticipation of loss, alongside a sense of relief related to the end of the care recipient’s suffering.

*(P10): “(…) It’s a very difficult feeling to explain… on one hand, it hurts so much to think I might lose her—just the thought makes me deeply sad. But then there are moments when I feel that, if it happens, at least she would no longer suffer, and that brings me some relief… and I even feel guilty for thinking that way. When she really starts to suffer, may God take her—I don’t want to see her suffer.”*

*(P9): “(…) if he passes, I don’t want him to suffer. If he were in a lot of pain, I think it would be a relief.”*

*(P1): “(…) death is a relief, because it’s not easy to watch someone deteriorate—it’s very sad.”*

Thus, caregivers remain in an intermediate space marked by uncertainty and the anticipation of the end. A space wherein the bond persists but is continuously permeated by the awareness of its imminent loss.

#### 3.2.2. Coping with Challenges in the Care Relationship

Challenges in the care relationship with the ill person constitute a relevant dimension of caregivers’ experiences, reflecting the complexity of relational dynamics inherent in caregiving. In this context, five subthemes emerged: (1) asymmetry in the care relationship; (2) the familial relationships replaced by the caregiving relationships; (3) tension between obligation and a sense of mission; (4) denial and avoidance; and (5) confrontation with human fragility.

(1) Asymmetry in the care relationship

Asymmetry in the care relationship emerges as a central element, highlighting the reconfiguration of roles between caregiver and care recipient. As the illness progresses, the relationship tends to become unequal, marked by greater control and decision-making on the part of the caregiver, which may alter relational balance and reduce the autonomy of the ill person.

*(P1): “At the beginning, we could still decide things together… but over time I realized that he no longer had that capacity. Now I have to decide everything, from simple things to the most important ones. Sometimes I feel like I’ve gone from being a wife to someone who directs, who controls… and that’s hard for me, because I can see he no longer has the same autonomy—he depends on me for almost everything.”*

*(P11): “Above all, I want to make sure everything is under control so that nothing goes wrong (…).”*

(2) Familial relationships replaced by the caregiving relationships

The replacement of the familial relationship by the caregiving relationship emerges as a significant transformation in relational dynamics, reflecting the progressive loss of affective and intimate dimensions typical of a couple’s relationship. As the illness advances, interactions become predominantly centered on caregiving tasks, leading to a redefinition of roles and a dilution of marital identity.

*(P5): “I feel that, little by little, we have stopped being a couple as we once were. There is no longer space for the things that used to connect us, like calm conversations or moments of closeness… now almost everything revolves around care.”*

*(P1): “Our relationship as a couple has practically ended. We no longer have a marital relationship.”*

(3) Tension between obligation and a sense of mission

The tension between obligation and a sense of mission emerges as a significant dimension of caregivers’ experiences, reflecting the coexistence of a moral duty to care and the attribution of meaning to that role. The accounts reveal a strong emotional and relational commitment to the care recipient, in which caregiving is perceived both as an assumed responsibility and as a continuation of prior relationships, providing meaning, purpose, and integration within caregivers’ life trajectories.

*(P11): “If she cared for me for 50 years, am I going to abandon her now, when she needs me most? (…) I feel that I have an obligation to support her.”*

*(P2): “(…) deep down, I’ve always been a caregiver. It’s my mission (…) from a very early age I started caring. I took care of my grandmother, an uncle, another grandmother, my father, and now my mother.”*

*(P3): “I’ve always been someone who adapts easily to these situations because I cared for my mother (…) then my mother-in-law became bedridden, and I was the one who took care of her (…) I adapted very easily, because this is the cycle of life (…).”*

(4) Denial and avoidance

Denial and avoidance emerge as ways of managing the caregiving experience, reflecting a tendency to avoid thinking about the progression of the situation and the inevitability of death. Participants describe different forms of distancing, including postponing reflection and minimizing the severity of the situation.

*(P8): “Right now, I avoid thinking about what will happen, because it makes me feel overwhelmed. I don’t want to think that death might be imminent.”*

*(P5): “(…) I know it might be wrong, what I’m thinking, but I feel that it’s still something far off, so I don’t want to give it too much importance (…).”*

*(P15): “I can’t imagine myself without him, but I know that sooner or later it will happen. It’s very difficult—and even more so because he is very aware of what is happening, which makes everything even harder.”*

(5) Confrontation with human fragility

Awareness of human fragility becomes evident in the caregiving experience as participants describe the progressive physical deterioration of the care recipient. They report marked limitations in mobility and communication, reflecting an increasing dependency and need for support in basic activities.

*(P14): “(…) things have been getting more difficult because she is losing more and more mobility. She needs my help (…).”*

*(P10): “She doesn’t speak, she doesn’t move—it’s just the body there, she is trapped in her own body.”*

*(P7): “Her condition is degenerative, so day by day you can see increasing dependency (…) then the inability to move—legs, arms, even the head (…) so less and less mobility, with greater and greater dependence.”*

*(P15): “It’s deeply impactful to see someone who was always very active become completely dependent (…) when my father is worse, with nausea, vomiting, and constant complaints—those are the moments that are hardest for me to deal with.”*

Thus, physical fragility emerges as a factor that not only intensifies the practical demands of caregiving but also reconfigures relational balance, shifting it from a logic of reciprocity to one marked by dependency.

#### 3.2.3. Assigning Meaning and Purpose to the Anticipation of Death

Living with meaning and purpose in the face of a loved one’s anticipated death constitutes a relevant dimension of caregivers’ experiences, reflecting how they seek to emotionally integrate the impending loss and attribute meaning to the caregiving process. In this context, four subthemes emerged: (1) dignity-preserving actions; (2) living one day at a time; (3) faith and spirituality; and (4) specialized support.

(1) Dignity-preserving actions

Dignity-preserving actions emerge as a central strategy in caregivers’ experiences, reflecting an ongoing concern to maintain the well-being, autonomy, and functional integrity of the care recipient. This concern is expressed through the promotion of activities and stimulation of remaining abilities, as a way of counteracting the decline associated with disease progression.

*(P11): “(…) whenever I go out, she always comes with me. I make a point of taking her so she can get out of the house and get some fresh air.”*

*(P9): “At home, I try to encourage mobility whenever I see that it’s appropriate (…) so that he doesn’t lose it completely.”*

*(P1): “At the moment, I can only go for short walks with my husband (…) I usually take him by car on Fridays or Saturdays, when he has a bit more strength to walk.”*

Taken together, these accounts show that caregiving goes beyond instrumental tasks, becoming a practice oriented toward safeguarding dignity. Even in the face of progressive fragility, caregivers strive to preserve a sense of autonomy, identity, and continuity through small actions that resist the advance of dependency.

(2) Living one day at a time

A focus on the present daily routine emerges as an adaptive strategy among caregivers, a way of dealing with uncertainty linked to disease progression. Participants’ accounts indicate a pragmatic acceptance of their situation, emphasizing “living one day at a time” as an emotional adjustment mechanism that reduces future-oriented thinking and promotes a sense of control and stability in the face of caregiving demands.

*(P2): “(…) I’ve always had the idea that we have to live one day at a time and make the most of the opportunities that come our way.”*

*(P7): “There’s nothing we can do. The disease is incurable, so there’s no point in fighting it because it’s impossible. We just try to adapt and live one day at a time, as best as we can.”*

*(P5): “(…) I prefer to live day by day and do the best I can to ensure his comfort.”*

(3) Faith and spirituality

Faith and spirituality emerge as significant dimensions in caregivers’ experiences, reflected in the use of religious beliefs as a means of acceptance and meaning-making in the face of disease progression. The narratives reveal attitudes of surrender and trust in a higher power, functioning as coping mechanisms that help manage uncertainty, foster acceptance, and provide emotional support.

*(P10): “(…) at the beginning, I was discouraged and cried a lot. Then I had to accept that life is like this… as long as God wills it, this will be my life (…). It is in God’s hands.”*

*(P6): “I truly believe there is a purpose in all of this… sometimes that belief helps me accept what we are going through. I place everything in God’s hands, asking for strength to keep going and for him not to suffer so much.”*

*(P8): “My future belongs to God—today is like this, and tomorrow we will see. There are things I know I cannot control, and faith gives me some peace, as if I’m not alone in this.”*

(4) Specialized support in legitimizing pain before loss

Specialized support emerges as an important resource in caregivers’ experiences, reflecting the value of support from healthcare professionals in emotional sharing, validation of caregiving practices, and management of caregiving demands. By providing guidance, attentive listening, and emotional containment, healthcare professionals help caregivers integrate anticipatory grief into their daily lives, enabling gradual adaptation and preparing them more effectively for the eventual loss.

*(P14): “I have the support of the palliative care team. With this support, I am slowly preparing myself for what I know may happen. It doesn’t solve everything, but it helps me not to be completely caught off guard.”*

*(P1): “The support I’ve received makes a huge difference because I feel that someone understands what I’m going through. Talking about it makes me feel less alone in this process, which is already so heavy.”*

*(P5): “(…) talking to the nurse or the psychologist helps me (…) because there are things I already feel, as if I were grieving ahead of time.”*

Specialized support fosters a sense of being understood and accompanied, contributing to greater emotional resilience and facilitating a more adaptive coping process in the face of impending loss.

## 4. Discussion

To the best of our knowledge, this study represents the first effort in Portugal to examine the experience of anticipatory grief among family caregivers of individuals receiving PC in a home setting. The findings suggest that anticipatory grief is a process of meaning reconstruction in the face of imminent loss, reflected in caregivers’ efforts to balance the demands of caregiving with the emotional impact of progressive loss, adapt to changing relationship dynamics and roles, and develop coping strategies that facilitate emotional adjustment to the anticipated death of a loved one. Together, these experiences illustrate anticipatory grief as a dynamic process of both distress and adaptation.

The findings indicate that the experience of anticipatory grief is configured as a process intrinsically linked to the demands of caregiving, manifested as progressive and significant transformations in caregivers’ daily lives [21,43]. The reorganization of routines, restriction of personal activities, and loss of autonomy emerge as central categories, highlighting the impact of continuous caregiving on individual and social domains. These elements are consistent with the literature, which points to a transversal impact of caregiving across personal, social, and professional dimensions, associated with high levels of burden [44].

Within this framework, caregiver burden emerges as a structuring dimension of anticipatory grief, expressed through physical and emotional exhaustion, persistent worry, and impairment of overall well-being. The intensity of caregiving demands, combined with prolonged exposure to the deterioration of the care recipient, contributes to increased stress and reduced quality of life [44,45]. Concomitantly, there is a tendency to devalue one’s own needs, particularly in contexts of limited social support, which may intensify experiences of isolation and vulnerability [46,47]. Nevertheless, the appreciation of small moments of self-care emerges as a relevant emotional regulation strategy, reflecting adaptive processes.

Caregivers’ emotional experience is, in turn, marked by inherent ambivalence. Sadness related to the anticipation of loss coexists with feelings of relief regarding the potential cessation of the care recipient’s suffering. This coexistence of seemingly contradictory emotions emerges as a central characteristic of anticipatory grief, reflecting its dynamic nature and the accumulation of progressive losses throughout the course of the illness [43,48].

Concurrently, anticipatory grief is embedded in a process of transformation of relational and identity dynamics. Disease progression leads to the redefinition of roles and the emergence of relationships marked by asymmetry, often characterized by a transition from previously reciprocal bonds to relationships centered on caregiving. This process may entail a weakening of affective dimensions due to the predominance of instrumental functions [44]. However, caregiving is simultaneously sustained by a strong sense of commitment and relational continuity, and is experienced both as an obligation and as an expression of emotional attachment, thus constituting a process of meaning-making in the face of imminent loss [21,22].

Within this processual context, caregivers mobilize different emotional regulation strategies in response to uncertainty and disease progression. Avoidance and denial emerge as psychological protective mechanisms that, while temporarily reducing emotional impact, may hinder the grieving process in the long term. This dynamic is consistent with conceptualizations of grief as a non-linear process, marked by oscillations between confrontation and avoidance [6]. The growing awareness of human fragility and finitude intensifies psychological distress, requiring the mobilization of adaptive resources. Among these, a present-oriented perspective—expressed as “living one day at a time”—stands out as a strategy that facilitates the management of uncertainty and promotes emotional adjustment [24]. Spirituality and faith also emerge as meaningful resources for meaning-making and acceptance, contributing to caregivers’ resilience [23,49]. Additionally, concern with preserving the dignity of the care recipient reflects the adoption of a person-centered approach, aligned with the principles of PC, which prioritizes well-being and quality of life until the end of life [4,50,51]. Finally, specialized support emerges as a transversal facilitator throughout the entire process. Support from healthcare professionals proves fundamental not only in providing care to the patient but also in supporting caregivers, promoting adaptation to caregiving demands, reducing burden, and enhancing psychological well-being throughout the anticipatory grief process [25,27,52].

Overall, the findings highlight anticipatory grief as an integral component of the caregiving experience in home-based palliative care. The emotional, relational, and practical challenges identified reinforce that community PC services need to adopt a proactive approach toward caregivers’ psychosocial needs [53,54]. Early identification of anticipatory grief, together with interventions aimed at strengthening coping resources, social support, and meaning-making processes, may help mitigate caregiver burden and promote psychological adjustment throughout the illness trajectory and into bereavement [55].

### 4.1. Study Strengths and Limitations

The use of CGT proved particularly appropriate for exploring the experience of anticipatory grief, allowing for an in-depth, processual, and contextualized understanding of caregivers’ experiences. Conducting semi-structured interviews enabled access to detailed and reflective narratives, supporting the exploration of emotional, relational, and meaning-making dimensions of the experience. In several cases, the interviews represented rare opportunities to verbalize experiences and emotions associated with caregiving, within a daily context marked by constant demands and limited recognition of their role. In this sense, the interview setting may have facilitated processes of emotional validation by providing a space for attentive and nonjudgmental listening. Additionally, the progressive inclusion of participants based on emerging analytical needs contributed to the refinement of categories and to the conceptual density of the developed model.

However, several limitations were identified. First, recruitment within a single region may limit the transferability of the findings to other cultural, institutional, or geographical contexts. Furthermore, restricting the sample to family caregivers of cancer patients excludes those caring for individuals with other conditions, thereby potentially limiting the broader applicability of the findings to different caregiving populations or to those providing care in inpatient or long-term care settings. In addition, the absence of detailed care-recipient characteristics may have restricted a fuller understanding of the caregiving context and its influence on grief experiences. Future research should incorporate these variables to better examine how caregiving circumstances may shape caregivers’ experiences of grief. Second, addressing a sensitive and emotionally demanding topic such as anticipatory grief may have influenced the degree of openness and spontaneity in participants’ accounts, particularly in the early stages of engagement. Moreover, some participants exhibited a tendency to avoid directly addressing the topic, a finding that is consistent with the nature of the phenomenon, often characterized by avoidance mechanisms in response to the perception of imminent loss. Finally, identifying participants through healthcare teams may have favored the inclusion of caregivers who are more engaged or more connected to services, potentially overlooking more marginalized or less visible experiences. Future mixed-method and longitudinal studies following caregivers over time (during illness and after death) may provide further insights into the evolution of anticipatory grief and its transition into post-loss grief. Additionally, future research may benefit from triangulating perspectives by including not only family caregivers but also healthcare professionals and, when possible, individuals receiving PC themselves. Such an approach would enable a more comprehensive and integrated understanding of anticipatory grief, allowing for the analysis of convergences and divergences in experiences and meanings, thereby contributing to both theoretical advancement and the development of more tailored interventions.

### 4.2. Implications for Practice

The findings highlight the need to strengthen the integration of family caregivers as a central focus in PC practice, as their needs often remain unmet, particularly at the emotional, informational, and practical levels [56]. In this context, the training and preparation of healthcare professionals emerge as a key structural element, particularly in EoL communication, which is crucial for the early identification of needs and for delivering more effective, family-centered care [57].

Investing in both initial and ongoing training for healthcare professionals, with a particular focus on understanding anticipatory grief as a complex and often undervalued process, is also essential. Integrating this domain into educational curricula and professional development programs can enhance clinical sensitivity, enabling professionals to recognize early signs of emotional distress in caregivers and to implement more appropriate interventions throughout the illness trajectory.

In this regard, several best practices can be highlighted: (1) conducting regular assessments of caregivers’ needs, including systematic screening for emotional distress and signs of anticipatory grief; (2) promoting spaces for active listening and emotional validation through caregiver-focused consultations; (3) involving caregivers in clinical decision-making and ensuring clear, gradual, and tailored communication; (4) developing psychoeducational programs addressing the illness trajectory and anticipatory grief; and (5) facilitating access to specialized psychological support whenever necessary.

Additionally, it is imperative to develop and implement structured interventions targeted at caregivers, grounded in scientific evidence and supported by health policies that formally recognize their role and ensure continuous support, both during the illness and throughout the bereavement period [26]. The adoption of integrated care models, based on multidisciplinary and longitudinal approaches, also plays a central role, contributing to reducing caregiver burden and improving health outcomes [58].

Within this framework, structured emotional support and psychosocial interventions are essential, not only for reducing psychological distress but also for facilitating adjustment to anticipatory grief and promoting caregivers’ overall well-being [59,60]. Therefore, the systematic incorporation of these dimensions into clinical practice represents a fundamental step toward a more humanized, preventive, and caregiver-centered approach.

## 5. Conclusions

This study, rooted in Charmaz’s CGT, enabled an understanding of anticipatory grief among family caregivers of individuals receiving PC at home as a dynamic process of (re)constructing meanings in the face of imminent loss within the caregiving context. This central category highlights the processual, subjective, and relational nature of the experience, in which caregivers seek to make sense of the progressive deterioration of the care recipient and the imminence of their loss. Caregivers experience a continuous tension between maintaining everyday life and confronting the anticipated loss of a loved one, while simultaneously adapting to changing relational roles and increasing caregiving demands.

The study further highlights that anticipatory grief is characterized not only by suffering and emotional burden but also by ongoing efforts to find meaning, preserve relational bonds, and mobilize personal and social resources that support adaptation. Meaning-making, spirituality, social support, and professional support emerged as important facilitators in managing the uncertainty and emotional demands associated with caregiving and impending loss. These findings reinforce the view of anticipatory grief as a multidimensional and adaptive process, rather than merely a precursor to bereavement. Overall, the study underscores the need for palliative care services to recognize anticipatory grief as a central component of the caregiving experience and to provide proactive, family-centered interventions that support caregivers’ emotional well-being, coping capacity, and adaptation throughout the illness trajectory.

## Figures and Tables

**Table 1:** Background of participants (N = 15).

Participant	Age	Gender	Marital Status	Education Level	Employment Status	Relationship to Care Recipient	Lives with Care Recipient?	Duration of Care (Months)	Family Support Network?	Social Support Network?
P1	66	Female	Married	Higher Education	Retired	Spouse	Yes	≥12	Yes	Yes
P2	66	Female	Married	Higher Education	Full-time employment	Daughter	No	6–12	Yes	No
P3	67	Female	Married	Lower Secondary	Retired	Spouse	Yes	≥12	Yes	Yes
P4	74	Male	Married	Primary Education	Retired	Spouse	Yes	6–12	Yes	No
P5	71	Female	Married	Higher Education	Retired	Spouse	Yes	≥12	Yes	Yes
P6	74	Female	Married	Primary Education	Retired	Spouse	Yes	≥12	Yes	Yes
P7	70	Male	Married	Secondary Education	Retired	Spouse	Yes	≥12	No	Yes
P8	65	Female	Married	Lower Secondary	Full-time employment	Spouse	Yes	≥12	No	Yes
P9	71	Female	Married	Higher Education	Retired	Spouse	Yes	≥12	Yes	Yes
P10	71	Female	Married	Primary Education	Retired	Daughter	Yes	≥12	Yes	Yes
P11	76	Male	Married	Secondary Education	Retired	Spouse	Yes	≥12	Yes	Yes
P12	79	Female	Married	Primary Education	Retired	Spouse	Yes	≥12	Yes	No
P13	74	Female	Widowed	Primary Education	Retired	Daughter	Yes	≥12	No	Yes
P14	71	Female	Single	Secondary Education	Retired	Daughter	Yes	≥12	No	No
P15	63	Female	Married	Higher Education	Full-time employment	Father	Yes	≥12	Yes	No

## Data Availability

The raw data supporting the conclusions of this article will be made available by the authors on request. This article is based on the first author’s master’s dissertation in Palliative Care at the School of Health Sciences—Polytechnic University of Leiria.

## Supplementary Materials

The following supporting information can be downloaded at https://www.mdpi.com/article/10.3390/healthcare14152312/s1, Table S1: Consolidated criteria for reporting qualitative studies (COREQ): 32-item checklist.

## Abbreviations

The following abbreviations are used in this manuscript:

CGT	Constructivist Grounded Theory
PC	Palliative Care

## Appendix A

**Table A1:** Interview Guide.

Topic	Questions
Caregiving Experience	1. I would like you to tell me about how you have been experiencing accompanying [name of the person] at this stage of life. 1.1. What is a typical day like for you? 1.2. What changes have you noticed in your life since the illness began? 2. Has there been any particularly significant or difficult moment during this process that you would like to share? 2.1. How did you cope with that situation?
Relationship with the Person in Palliative Care	3. How would you describe your relationship with [name of the person] during this time? Have you noticed any changes? 3.1. What do you feel has remained the same, and what has changed? 3.2. What impact has this had on you? 4. Do you notice differences compared to your prior relationship? In what ways?
Anticipation of Loss	5. Have you thought about the possibility of losing [name of the person]? How have you been experiencing this? 5.1. What feelings arise when you think about it? 5.2. Is there anything you try to avoid thinking about or feeling?
Needs and Support	6. What do you feel you need most, or what has been missing, in order to cope with all of this? 6.1. What kind of help or support would you like to have?
Personal Changes	7. Do you feel that this experience has changed you? In what ways do you feel you are different now compared to before? 7.1. Has it changed the way you see life, other people, or yourself? 8. In what way has your perspective on the future changed? 8.1. Despite everything that is happening, do you feel that your life still has meaning? What gives your life meaning?
Conclusion	9. Is there anything we have not yet discussed that you consider important to add?
